# Neoadjuvant Chemotherapy Versus Primary Cytoreductive Surgery for Metastatic Endometrial Cancer

**DOI:** 10.1002/cam4.71539

**Published:** 2026-01-20

**Authors:** Dib Sassine, Yongmei Huang, Chin Hur, Elena B. Elkin, Jennifer S. Ferris, Alex Melamed, Chung Yin Kong, Evan R. Myers, Nina A. Bickell, William D. Hazelton, Tracy M. Layne, Brandy Heckman‐Stoddard, Goli Samimi, Laura J. Havrilesky, Stephanie V. Blank, Xiao Xu, Jason D. Wright

**Affiliations:** ^1^ Department of Obstetrics and Gynecology Columbia University College of Physicians and Surgeons New York New York USA; ^2^ Department of Medicine Columbia University College of Physicians and Surgeons New York New York USA; ^3^ Herbert Irving Comprehensive Cancer Center New York New York USA; ^4^ Joseph L. Mailman School of Public Health Columbia University New York New York USA; ^5^ Department of Obstetrics and Gynecology Massachusetts General Hospital Boston Massachusetts USA; ^6^ Division of General Internal Medicine Icahn School of Medicine at Mount Sinai New York New York USA; ^7^ Department of Obstetrics and Gynecology at the Duke University Medical Center Durham North Carolina USA; ^8^ Department of Population Health Science and Policy Icahn School of Medicine at Mount Sinai New York New York USA; ^9^ Herbold Computational Biology Program, Division of Public Health Sciences Fred Hutchinson Cancer Center Seattle Washington USA; ^10^ Division of Cancer Prevention National Cancer Institute Bethesda Maryland USA

## Abstract

**Objective:**

To evaluate the pattern of use and clinical outcomes associated with neoadjuvant chemotherapy (NACT) compared with primary debulking surgery (PDS) in patients with stage IV endometrial cancer.

**Methods:**

We utilized the National Cancer Database to identify individuals diagnosed with stage IV endometrial cancer, and categorized them according to receipt of NACT or PDS. Propensity score weighting using inverse probability of treatment weighting was applied. Survival outcomes were evaluated using both an intention‐to‐treat (ITT) analysis, which included all eligible patients, and a per‐protocol (PP) analysis restricted to those who underwent chemotherapy and surgery.

**Results:**

Among 18,205 patients, NACT utilization rose from 30.3% in 2010 to 73.8% in 2021 (*p* < 0.0001). In the multivariable analysis, patients diagnosed in more recent years, Black and Hispanic race and ethnicity, Medicaid insurance, serous histology, and greater comorbidities were associated with NACT (*p* < 0.05). In the ITT analysis, there was no mortality difference within 4 months after diagnosis between NACT patients and PDS patients (aHR = 1.03; 95% CI: 0.96–1.11); however, after 4 months, patients treated with NACT experienced higher mortality than those undergoing PDS (aHR = 1.58; 95% CI: 1.51–1.64). In the PP analysis, NACT patients had lower mortality compared to PDS patients within 24 months after diagnosis (aHR = 0.93; 95% CI, 0.88–0.99) but a 34% higher mortality after 24 months (aHR = 1.34; 95% CI, 1.23–1.47).

**Conclusion:**

Utilization of NACT has expanded among patients with metastatic endometrial cancer. Primary debulking surgery with postoperative chemotherapy is linked to higher early mortality but improved long‐term outcomes relative to treatment strategies beginning with NACT followed by surgery.

## 1 Introduction

Endometrial cancer represents the most frequently diagnosed gynecologic cancer in the United States. In 2024, an estimated 67,880 new cases and 13,250 deaths are anticipated [1]. While most individuals present with early‐stage, uterine‐confined disease, approximately 10%–20% of patients are diagnosed at an advanced stage [2, 3], which is responsible for more than half of the deaths attributed to endometrial cancer [4].

Historically, treatment for stage IV endometrial cancer was surgical cytoreduction followed by chemotherapy [5]. Primary debulking surgery (PDS) aims to remove all gross disease. Primary cytoreductive surgery is associated with substantial morbidity, often requires extensive upper abdominal surgery, and results in gross residual disease in a significant number of patients despite maximum surgical effort [5]. As an alternative approach for primary cytoreduction, neoadjuvant chemotherapy (NACT) with subsequent interval cytoreduction has been introduced [5, 6, 7].

NACT aims to reduce tumor volume prior to surgery. Although no randomized trials exist to compare NACT and primary cytoreduction, prior work suggests that NACT utilization for stage IV endometrial cancer is rising [6]. The impact of use of NACT on survival for metastatic endometrial cancer remains uncertain with some retrospective studies showing similar survival, while other data have suggested that long‐term survival is superior for primary cytoreduction [8]. We previously observed that, in the period immediately following diagnosis, treatment with NACT was associated with better survival than PDS, but for longer term survivors, outcomes were superior for primary cytoreduction [6].

Given the increasing utilization of NACT in patients diagnosed with advanced stage endometrial cancer in the U.S. and the availability of more recent data, our study aimed to examine survival for NACT and primary cytoreduction. Specifically, we examined contemporary patterns of care and short‐ and long‐term survival among patients with stage IV endometrial cancer treated with NACT or primary cytoreduction.

## 2 Methods

### 2.1 Study Design and Data Source

We conducted our analysis using the National Cancer Database (NCDB), a collaboration between the American College of Surgeons Commission on Cancer and the American Cancer Society, which focuses on comprehensive oncology services and outcomes [9]. The NCDB includes data from more than 1500 hospitals associated with the Commission on Cancer (CoC) in the U.S. and captures roughly 70% of all newly incident cancer cases nationwide. It provides detailed information about individual demographics, profiles of facilities providing cancer care, tumor characteristics, initial treatments, short‐term mortality, and long‐term survival data. The CoC maintains stringent quality assurance processes, including regular audits. As the NCDB does not contain identifiable patient information, the Columbia University Institutional Review Board classified this study as nonhuman subjects research, and informed consent was not required.

### 2.2 Cohort Selection

Our study cohort consisted of patients diagnosed with endometrial cancer during the years 2010 through 2021. The study cohort was restricted to stage IV patients with endometrioid, serous, clear cell, and carcinosarcoma histologies. We excluded patients without endometrial cancer as a first primary disease, diagnosis not confirmed pathologically, unknown status of chemotherapy, undetermined sequence between chemotherapy and PDS, and having chemotherapy and PDS initiated on the same day. The final trend analysis and survival cohorts included 18,205 patients and 16,450 patients, respectively.

### 2.3 Primary Treatment and Survival Outcomes

NCDB provides information for the first course of treatments. We defined primary treatment based on days from diagnosis to definitive surgery and initiation of chemotherapy. Patients were classified as receiving NACT if the first treatment was chemotherapy within 90 days of diagnosis regardless of subsequent surgery. Patients were categorized as receiving PDS if the first therapy was a hysterectomy or exenteration. These two treatment groups were analyzed in the intention‐to‐treat (ITT) analysis. We subsequently documented receipt of hysterectomy after NACT and receipt of chemotherapy after hysterectomy during the first course of treatment. Given that the combination of chemotherapy and surgery is preferred management for stage IV endometrial cancer, the per‐protocol (PP) cohort was restricted to patients who underwent both treatments.

The primary long‐term outcome was all‐cause mortality, calculated as the number of months between diagnosis and either last follow‐up or death. The survival probabilities and restricted mean survival time (RMST) were measured at 1‐, 2‐, and 5‐year. The short‐term mortality outcomes included mortality cases at 30, 90, 120, and 180 days after primary treatment per 100 patients.

### 2.4 Covariates

Demographic and clinical variables included age at diagnosis (< 40, 40–49, 50–59, 60–69, 70–79, ≥ 80 years), race and ethnicity (Non‐Hispanic White, Non‐Hispanic Black, Non‐Hispanic other, Hispanic, unknown), insurance status (uninsured, private, Medicaid, Medicare, other government, unknown), zip‐code neighborhood median household income (< $46,277, $46,277–$57,856, $57,857–$74,062, > $74,062), area of residence (metropolitan, urban, rural, unknown), year of diagnosis, and Charlson comorbidity score (0, 1, 2 or higher). Cancer‐related characteristics included stage (stage IVA—local extension to the rectum or bladder, stage IVB—distant metastases; IV not otherwise specified [NOS]), histologic type (endometrioid, serous, clear cell, carcinosarcoma, endometrial NOS), and tumor grade (well differentiated, moderately differentiated, poorly differentiated, undifferentiated, or unknown). Facility factors encompassed geographic region (Northeast, South, Midwest, West) and facility type (community cancer program, comprehensive community cancer program, academic and research program, integrated network cancer program).

### 2.5 Statistical Analysis

Patient demographics, clinical factors, cancer characteristics, and facility factors are presented descriptively and stratified based on primary treatment with NACT or PDS. Trends in the utilization of NACT were examined using Cochran‐Armitage Trend tests in the overall ITT cohort, including patients diagnosed from 2010 to 2021. Trends in four treatment subcategories (chemotherapy followed by surgery, chemotherapy alone, surgery followed by chemotherapy, surgery alone) are presented. We leveraged a random effects binomial logistic regression model to examine factors associated with the utilization of NACT in the ITT cohort accounting for hospital clustering. Adjusted odds ratios (aOR) and 95% confidence intervals (CI) were reported from this random effects logistic regression model.

We performed a propensity score (PS) analysis to compare survival NACT‐ and PDS‐treated patients in the ITT and PP cohorts from 2010 to 2020. Patients diagnosed in 2021 were excluded in the survival analysis because survival information was not available. PSs were derived from the predicted likelihood of receiving NACT based on pretreatment baseline factors prior to treatment [10], including age at diagnosis, race and ethnicity, health insurance, neighborhood median household income, urban/rural location, year of cancer diagnosis, comorbidity score, facility location, facility type, cancer stage, histologic type, and tumor grade.

The inverse probability of treatment weighting (IPTW) technique was used to establish a pseudo study cohort where the distribution of baseline factors was balanced between treatment groups [10]. The weight for each patient was assigned as the inverse predicted probability for each treatment arm. That is, the weights for patients in the NACT group equal 1/predicted probability of NACT; while the weights for patients in the PDS group equal 1/(1‐predicted probability of NACT) [11]. Stabilized IPTW weights were used to reduce variance, and weights greater than 10 were trimmed to limit the influence of outliers with extreme weights [11]. Standardized mean differences (SMD) were calculated for each baseline factor to assess the balance diagnostics in the original cohort and PS IPTW cohort. A SMD of less than 0.1 was considered well balanced between treatment arms [12].

Adjusted survival curves with inverse probability weights are presented [13]. All‐cause mortality at 30‐, 90‐, 120‐, and 180‐days and long‐term survival at 1‐, 2‐, and 5‐years were compared using IPTW survival curves. We noted a time‐varying association between primary treatment and survival. As survival curves crossed, we assessed survival before and after the crossing point. Given evidence of nonproportional hazards (crossing survival curves), we developed time‐varying Cox models in the PS‐IPTW cohort to compare overall mortality between NACT and PDS. We modeled time‐varying treatment effect by incorporating an interaction term between treatment and follow‐up time (months). After identifying the time point at which the hazard ratio changed direction (the curve‐crossing time, t*), we specified a piecewise model with a single knot at the crossing time (t*). The model yields period‐specific hazard ratios for time zero to the crossing time t* (0 − t*), and for the time beyond the crossing time t* (*t* > t*), respectively. The time‐varying Cox model was developed for all the stratification analysis. RMST was measured in the unweighted original cohort, and differences between treatment arms were evaluated using multivariable RMST regression, accounting for baseline factors including age at diagnosis, race/ethnicity, health insurance, neighborhood median household income, urban/rural location, year of cancer diagnosis, comorbidity score, facility location, facility type, cancer stage, histologic type, and tumor grade.

To examine the stability of our results across clinically relevant groups, stratified analyses were conducted among subgroups of patients (limited to patients younger than 70 years without any Charlson comorbidities, by cancer stage, and by histologic type, respectively). For each stratified analysis, we repeated the process described above to develop IPTW cohorts.

All statistical tests were two‐sided, with *p* < 0.05 considered statistically significant. Analyses were performed using SAS version 9.4 (SAS Institute Inc., Cary, NC, USA) and R, version 3.4.2 (R foundation for Statistical Computing, Vienna, Austria).

## 3 Results

**FIGURE 1:**
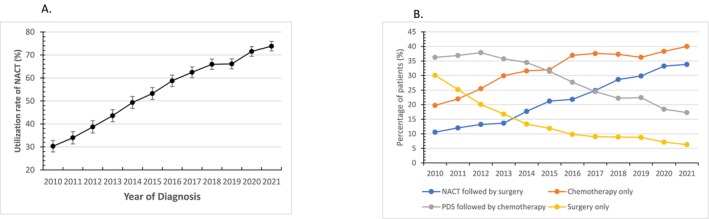
Utilization of neoadjuvant chemotherapy from 2010 to 2021. (A) Use of neoadjuvant chemotherapy. (B) Overall first course of treatment.

A total of 18,205 patients including 8064 (44.3%) who underwent PDS and 10,141 (55.7%) who received NACT were identified. Among patients who received NACT, 40.8% ultimately underwent surgery. For patients treated with PDS, 70.3% received postoperative chemotherapy. The use of NACT increased from 30.3% (95% CI, 27.9%–32.8%) in 2010 to 73.8% (95% CI, 71.7%–75.8%) in 2021 (*p* < 0.0001) (Figure 1).

The median age of diagnosis in both cohorts was 64 years (Table 1). In the multivariable regression model, patients diagnosed and treated in more recent years had substantially higher odds of receiving NACT (aOR for 2021 vs. 2010 = 6.66; 95% CI, 5.61–7.92). Compared with patients with private insurance, those with Medicaid had 41% higher odds of receiving NACT (aOR = 1.41; 95% CI, 1.25–1.60). Non‐Hispanic Black patients (aOR = 1.17; 95% CI, 1.07–1.29) and those of Hispanic ethnicity (aOR = 1.20; 95% CI, 1.04–1.39) were also more likely to receive NACT than non‐Hispanic White patients. While patients with two or more medical comorbidities were more likely to receive NACT (*p* < 0.0001), no association was observed between age at diagnosis and primary treatment allocation in the multivariable model. Overall, 51.8% of patients with endometrioid tumors received NACT, while 60.6% of those diagnosed with serous tumors received NACT and 46.4% of those with carcinosarcomas were treated with NACT (*p* < 0.0001).

**TABLE 1:** Factors associated with neoadjuvant chemotherapy in intention‐to‐treat cohort.

	Primary surgery	Neoadjuvant chemotherapy	*p*	aOR (95% CI)
	*N* (%)	*N* (%)		
Total	8064 (44.3)	10,141 (55.7)		
Age of diagnosis				
Median (IQR)	64 (58, 71)	64 (58, 71)	< 0.0001	
< 40	177 (41.4)	251 (58.6)		Referent
40–49	492 (46.1)	575 (53.9)		0.96 (0.72, 1.27)
50–59	1777 (45.6)	2117 (54.4)		0.94 (0.73, 1.21)
60–69	3184 (43.2)	4190 (56.8)		0.98 (0.76, 1.27)
70–79	1802 (43)	2388 (57)		1.03 (0.79, 1.34)
≥ 80	632 (50.5)	620 (49.5)		0.81 (0.61, 1.08)
Race/ethnicity				
Non‐Hispanic: White	5348 (45.8)	6318 (54.2)	< 0.0001	Referent
Non‐Hispanic: Black	1527 (40.0)	2288 (60.0)		1.17 (1.07, 1.29)*
Non‐Hispanic: Other	433 (47.3)	483 (52.7)		0.85 (0.73, 0.99)*
Hispanic	509 (38.4)	815 (61.6)		1.20 (1.04, 1.39)*
Unknown	247 (51)	237 (49)		0.99 (0.81, 1.23)
Year of diagnosis				
2010	938 (69.7)	408 (30.3)	< 0.0001	Referent
2011	796 (66.0)	410 (34.0)		1.22 (1.02, 1.46)*
2012	793 (61.3)	501 (38.7)		1.46 (1.23, 1.74)**
2013	776 (56.4)	600 (43.6)		1.78 (1.50, 2.11)**
2014	707 (50.7)	688 (49.3)		2.24 (1.89, 2.65)**
2015	659 (46.8)	750 (53.2)		2.61 (2.20, 3.09)**
2016	644 (41.2)	918 (58.8)		3.36 (2.84, 3.98)**
2017	605 (37.5)	1007 (62.5)		3.95 (3.34, 4.68)**
2018	567 (34.1)	1098 (65.9)		4.39 (3.71, 5.20)**
2019	622 (33.9)	1214 (66.1)		4.52 (3.83, 5.34)**
2020	498 (28.4)	1253 (71.6)		5.64 (4.76, 6.69)**
2021	459 (26.2)	1294 (73.8)		6.66 (5.61, 7.92)**
Insurance status				
Uninsured	342 (41.9)	474 (58.1)	< 0.0001	1.35 (1.13, 1.61)*
Private	3218 (46.6)	3687 (53.4)		Referent
Medicaid	639 (36.2)	1124 (63.8)		1.41 (1.25, 1.60)**
Medicare	3655 (44.3)	4596 (55.7)		0.99 (0.91, 1.08)
Other Government	67 (42.4)	91 (57.6)		1.31 (0.92, 1.88)
Unknown	143 (45.8)	169 (54.2)		0.92 (0.70, 1.21)
Neighborhood median household income				
< $46,277	1236 (41.2)	1765 (58.8)	< 0.0001	Referent
$46,277–$57,856	1476 (45.1)	1799 (54.9)		0.85 (0.76, 0.96)*
$57,857–$74,062	1784 (46.6)	2046 (53.4)		0.86 (0.76, 0.96)*
$74,063+	2504 (45.2)	3037 (54.8)		0.87 (0.78, 0.98)*
Unknown	1064 (41.6)	1494 (58.4)		0.90 (0.79, 1.03)
Urban/rural				
Metropolitan	6673 (44.0)	8476 (56.0)	0.4542	Referent
Urban	983 (45.3)	1185 (54.7)		0.97 (0.87, 1.09)
Rural	138 (47.3)	154 (52.7)		0.95 (0.73, 1.25)
Unknown	270 (45.3)	326 (54.7)		0.83 (0.68, 1.01)
Facility location				
Northeast	1707 (41.9)	2369 (58.1)	< 0.0001	Referent
South	2301 (48.3)	2462 (51.7)		0.85 (0.72, 1.00)*
Midwest	2575 (42.3)	3515 (57.7)		1.03 (0.89, 1.21)
West	1304 (45.8)	1544 (54.2)		0.84 (0.70, 1.00)*
Unknown	177 (41.4)	251 (58.6)		Non‐estimable
Facility type				
Community cancer program	270 (40.5)	396 (59.5)	< 0.0001	1.17 (0.94, 1.45)
Comprehensive community cancer program	2767 (47.7)	3031 (52.3)		0.95 (0.83, 1.09)
Academic/research program	3193 (41.0)	4587 (59)		Referent
Integrated network cancer program	1657 (46.9)	1876 (53.1)		0.95 (0.81, 1.10)
Other or unknown	177 (41.4)	251 (58.6)		1.00 (1.00–1.00)
Cancer stage				
IV NOS	507 (56.8)	385 (43.2)	< 0.0001	1.18 (0.97, 1.43)
IVA	687 (50.4)	677 (49.6)		Referent
IVB	6870 (43.1)	9079 (56.9)		1.00 (0.89, 1.14)
Histologic type				
Endometrioid	2900 (48.2)	3118 (51.8)	< 0.0001	Referent
Serous	2014 (39.4)	3092 (60.6)		1.19 (1.08, 1.31)*
Clear cell	316 (46.5)	363 (53.5)		0.81 (0.67, 0.97)*
Carcinosarcoma	1593 (53.6)	1379 (46.4)		0.60 (0.54, 0.67)**
Endometrioid NOS	1241 (36.2)	2189 (63.8)		1.67 (1.51, 1.85)**
Tumor grade				
Well	495 (50.8)	479 (49.2)	< 0.0001	Reference
Moderate	931 (50.1)	929 (49.9)		1.13 (0.95, 1.34)
Poorly	4034 (52.7)	3614 (47.3)		0.99 (0.85, 1.15)
Undifferentiated	750 (53.3)	657 (46.7)		1.21 (1.00, 1.46)
Unknown	1854 (29.4)	4462 (70.6)		2.68 (2.28, 3.14)**
Charlson comorbidity score				
0	5966 (44.6)	7398 (55.4)		Referent
1	1598 (45.6)	1909 (54.4)		1.02 (0.94, 1.11)
≥ 2	500 (37.7)	836 (62.3)		1.16 (1.01, 1.32)*

*Note:* Random effect logistic regression model indicates significant variation in NACT rates across hospitals.

Abbreviation: NOS, not otherwise specified.

* *p* < 0.05.

** *p* < 0.0001.

In the original unweighted cohort, the pre‐treatment baseline factors were not balanced between treatment arms in both ITT and PP analysis cohorts (Table S1). Following PS IPTW, the distribution of baseline factors between PDS and NACT groups was well balanced in both ITT and PP analyses, with all covariates showing SMDs < 0.1 (Table 2). In the ITT analysis, 90‐day mortality was 9.8% (95% CI, 9.0%–10.5%) in those who received NACT compared to 10.5% (95% CI, 9.6%–11.4%) in those who had PDS (Table 3). Short‐term mortality at 30, 60, 120, and 180 days did not differ significantly between the NACT and PDS groups. In the PP analysis, NACT followed by surgery had significantly lower mortality rates compared to PDS followed by chemotherapy at 90 days: 0.3% (95% CI, 0.1%–0.5%) for NACT versus 2.0% (95% CI, 1.6%–2.5%) for PDS. This differential continued, and at 180 days mortality was 2.3% (95% CI, 1.7%–2.9%) for those who received NACT and surgery compared to 8.0% (95% CI, 7.2%–8.8%) for those treated with PDS followed by chemotherapy. Similarly, in the subgroup analyses, NACT followed by surgery showed a significantly lower short‐term mortality compared to PDS followed by chemotherapy for patients aged under 70 years with no comorbidities, and for individual stages and histological subtypes (Table S2).

**TABLE 2:** Balance diagnostics for demographics and clinical factors in IPTW intention‐to‐treat cohort and per‐protocol cohort.

	Intention‐to‐treat analysis			Per‐protocol analysis		
	Primary surgery	Neoadjuvant chemotherapy	SMD	Primary surgery followed by chemotherapy	Neoadjuvant chemotherapy followed by surgery	SMD
	*N* (%)	*N* (%)		*N* (%)	*N* (%)	
Total	8780 (100%)	9889 (100%)		5191 (100%)	4141 (100%)	
Age of diagnosis						
< 40	239 (2.4)	212 (2.4)	0.04	104 (2.5)	126 (2.4)	0.008
40–49	580 (5.9)	520 (5.9)		258 (6.2)	319 (6.1)	
50–59	2162 (21.9)	1908 (21.7)		931 (22.5)	1160 (22.3)	
60–69	4016 (40.6)	3580 (40.8)		1782 (43.0)	2237 (43.1)	
70–79	2225 (22.5)	1966 (22.4)		876 (21.1)	1109 (21.4)	
≥ 80	668 (6.8)	595 (6.8)		190 (4.6)	240 (4.6)	
Race/Ethnicity						
Non‐Hispanic: White	6410 (64.8)	5686 (64.8)	0	2720 (65.7)	3407 (65.6)	0
Non‐Hispanic: Black	2041 (20.6)	1812 (20.6)		795 (19.2)	1008 (19.4)	
Non‐Hispanic: Other	485 (4.9)	440 (5.0)		237 (5.7)	294 (5.7)	
Hispanic	699 (7.1)	609 (6.9)		290 (7.0)	361 (6.9)	
Unknown	254 (2.6)	233 (2.7)		100 (2.4)	123 (2.4)	
Year of diagnosis						
2010	796 (8.1)	714 (8.1)	0	319 (7.7)	395 (7.6)	0
2011	715 (7.2)	643 (7.3)		286 (6.9)	369 (7.1)	
2012	798 (8.1)	702 (8.0)		341 (8.2)	416 (8.0)	
2013	837 (8.5)	739 (8.4)		346 (8.4)	427 (8.2)	
2014	847 (8.6)	756 (8.6)		367 (8.9)	459 (8.8)	
2015	840 (8.5)	740 (8.4)		364 (8.8)	461 (8.9)	
2016	934 (9.4)	832 (9.5)		385 (9.3)	487 (9.4)	
2017	970 (9.8)	872 (9.9)		394 (9.5)	498 (9.6)	
2018	999 (10.1)	891 (10.1)		419 (10.1)	528 (10.2)	
2019	1102 (11.1)	966 (11.0)		471 (11.4)	590 (11.4)	
2020	1051 (10.6)	926 (10.5)		447 (10.8)	560 (10.8)	
2021						
Insurance status						
Uninsured	455 (4.6)	394 (4.5)	0.05	165 (4.0)	207 (4.0)	0
Private	3795 (38.4)	3366 (38.3)		1741 (42.0)	2164 (41.7)	
Medicaid	963 (9.7)	864 (9.8)		364 (8.8)	455 (8.8)	
Medicare	4399 (44.5)	3910 (44.5)		1761 (42.5)	2232 (43.0)	
Other Government	91 (0.9)	81 (0.9)		47 (1.1)	57 (1.1)	
Unknown	187 (1.9)	164 (1.9)		64 (1.5)	77 (1.5)	
Neighborhood median household income						
< $46,277	1628 (16.5)	1441 (16.4)	0.03	633 (15.3)	803 (15.5)	0
$46,277–$57,856	1785 (18.0)	1581 (18.0)		743 (17.9)	927 (17.9)	
$57,857–$74,062	2116 (21.4)	1865 (21.2)		880 (21.2)	1102 (21.2)	
$74,063+	3009 (30.4)	2687 (30.6)		1314 (31.7)	1643 (31.6)	
Unknown	1351 (13.7)	1206 (13.7)		571 (13.8)	717 (13.8)	
Urban/rural						
Metropolitan	8208 (83.0)	7291 (83.0)	0	3431 (82.9)	4310 (83.0)	0
Urban	1204 (12.2)	1055 (12.0)		491 (11.9)	607 (11.7)	
Rural	152 (1.5)	140 (1.6)		67 (1.6)	89 (1.7)	
Unknown	325 (3.3)	294 (3.3)		151 (3.6)	185 (3.6)	
Facility location						
Northeast	2242 (22.7)	2010 (22.9)	0.07	931 (22.5)	1168 (22.5)	0
South	2602 (26.3)	2284 (26.0)		1087 (26.3)	1356 (26.1)	
Midwest	3273 (33.1)	2919 (33.2)		1376 (33.2)	1737 (33.5)	
West	1534 (15.5)	1355 (15.4)		643 (15.5)	805 (15.5)	
Unknown	239 (2.4)	212 (2.4)		104 (2.5)	126 (2.4)	
Facility type						
Community cancer program	369 (3.7)	322 (3.7)	0	104 (2.5)	130 (2.5)	0
Comprehensive community cancer program	3160 (32.0)	2784 (31.7)		1241 (30.0)	1561 (30.1)	
Academic/research program	4228 (42.8)	3778 (43.0)		1830 (44.2)	2289 (44.1)	
Integrated network cancer program	1893 (19.1)	1685 (19.2)		863 (20.8)	1086 (20.9)	
Other or unknown	239 (2.4)	212 (2.4)		104 (2.5)	126 (2.4)	
Cancer stage						
IV NOS	547 (5.5)	481 (5.5)	0.04	232 (5.6)	290 (5.6)	0
IVA	758 (7.7)	672 (7.6)		322 (7.8)	407 (7.8)	
IVB	8584 (86.8)	7628 (86.9)		3587 (86.6)	4495 (86.6)	
Histology type						
Endometrioid	3190 (32.3)	2822 (32.1)	0	1248 (30.1)	1572 (30.3)	0
Serous	2755 (27.9)	2489 (28.3)		1392 (33.6)	1752 (33.8)	
Clear cell	370 (3.7)	330 (3.8)		163 (3.9)	208 (4.0)	
Carcinosarcoma	1680 (17.0)	1485 (16.9)		710 (17.2)	869 (16.7)	
EM NOS	1894 (19.2)	1654 (18.8)		628 (15.2)	790 (15.2)	
Tumor grade						
Well	489 (4.9)	451 (5.1)	0	174 (4.2)	218 (4.2)	0.02
Moderate	1020 (10.3)	905 (10.3)		430 (10.4)	528 (10.2)	
Poorly	4175 (42.2)	3718 (42.3)		1920 (46.4)	2420 (46.6)	
Undifferentiated	871 (8.8)	770 (8.8)		420 (10.1)	518 (10.0)	
Unknown	3334 (33.7)	2935 (33.4)		1197 (28.9)	1507 (29.0)	
Charlson comorbidity score						
0	7258 (73.4)	6449 (73.4)	0	3081 (74.4)	3858 (74.3)	0
1	1925 (19.5)	1703 (19.4)		791 (19.1)	992 (19.1)	
2	457 (4.6)	406 (4.6)		177 (4.3)	224 (4.3)	
≥ 3	249 (2.5)	222 (2.5)		92 (2.2)	118 (2.3)	

*Note:* In the intention‐to‐treat analysis, the mean (SD) stabilized weights before the truncation was 1.15 (0.59) in the primary surgery arm and 1.20 (0.69) in the neoadjuvant chemotherapy arm. Weights exceeding 10 were truncated for 0 patients in the primary surgery arm and two patients in the neoadjuvant chemotherapy arm. In the per‐protocol analysis, the mean (SD) stabilized weights before the truncation was 1.12 (0.50) in the primary surgery arm and 1.22 (0.74) in the neoadjuvant chemotherapy arm. Weights exceeding 10 were truncated for 0 patients in the primary surgery followed by chemotherapy arm and two patients in the neoadjuvant chemotherapy followed by surgery arm.

Abbreviation: SMD, standardized mean difference.

**TABLE 3:** Adjusted short‐term mortality in intention‐to‐treat and per‐protocol analysis.

	IPTW intention‐to‐treat cohort		IPTW per‐protocol cohort	
	Neoadjuvant chemotherapy % (95% CI)	Primary surgery % (95% CI)	Neoadjuvant chemotherapy followed by surgery % (95% CI)	Primary surgery followed by chemotherapy % (95% CI)
30‐day	1.8 (1.5, 2.1)	2.0 (1.7, 2.4)	0.1 (0.0, 0.2)	0.1 (0.0, 0.2)
60‐day	5.9 (5.3, 6.5)	6.4 (5.7, 7.1)	0.1 (0.0, 0.3)	0.8 (0.6, 1.1)
90‐day	9.8 (9.0, 10.5)	10.5 (9.6, 11.4)	0.3 (0.1, 0.5)	2.0 (1.6, 2.5)
120‐day	13.2 (12.4, 14.1)	13.9 (12.9, 15.0)	0.7 (0.3, 1.0)	4.1 (3.5, 4.7)
180‐day	20.5 (19.4, 21.6)	18.8 (17.6, 20.0)	2.3 (1.7, 2.9)	8.0 (7.2, 8.8)

Abbreviation: CI, confidence interval.

**FIGURE 2:**
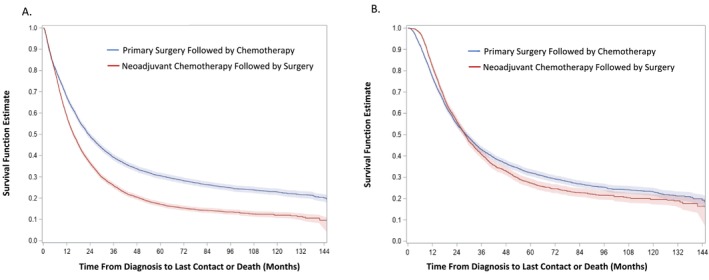
Inverse probability treatment weighting adjusted survival curves for overall cohort. (A) Intention‐to‐treat cohort. (B) Per‐protocol.

There was a time‐varying pattern of survival based on primary treatment (Figure 2, Table S3). In the ITT analysis, NACT and PDS patients had similar hazards of mortality during the first 4 months of diagnosis (aHR = 1.03; 95% CI, 0.96–1.11). Following 4 months, the survival curves crossed, and patients receiving NACT had a 58% higher risk of death compared with those undergoing PDS (aHR = 1.58; 95% CI, 1.51–1.64). Two‐year survival was 36.4% (95% CI, 35.1%–37.8%) for NACT groups versus 49.2% (95% CI, 47.7%–50.7%) for PDS group (Table 4). At 5 years, survival was 17.1% (95% CI, 16.0%–18.4%) versus 30.5% (95% CI, 29.1%–32.0%), respectively. In the PP analysis, NACT was associated with reduced mortality within the first 24 months of diagnosis (aHR = 0.93; 95% CI, 0.88–0.99). After 24 months, the receipt of NACT was associated with a 34% increase in mortality (aHR = 1.34; 95% CI, 1.23–1.47). Five‐year survival was 27.3% (95% CI, 25.3%–29.4%) for NACT versus 32.2% (95% CI, 30.5%–33.9%) for PDS. In the ITT analysis, the RMST for the NACT group was significantly lower than that for the PDS group at 12, 24, and 60 months after diagnosis (all *p* < 0.0001). In contrast, in the PP analysis, the NACT group had a significantly higher RMST than the PDS group at 12 and 24 months (both *p* < 0.0001), while there was no difference in the RMST at 60 months after diagnosis (Table 4).

**TABLE 4:** Adjusted long‐term survival rate in intention‐to‐treat and per‐protocol analysis.

	Intention‐to‐treat analysis		Per‐protocol analysis	
	Neoadjuvant chemotherapy	Primary surgery	Neoadjuvant chemotherapy followed by surgery	Primary surgery followed by chemotherapy
Survival (95% CI)				
1‐year	58.6 (57.4, 59.8)	67.5 (66.1, 69.0)	82.2 (80.6, 83.7)	77.2 (75.9, 78.5)
2‐year	36.4 (35.1, 37.8)	49.2 (47.7, 50.7)	56.2 (54.3, 58.3)	55.0 (53.4, 56.6)
5‐year	17.1 (16.0, 18.4)	30.5 (29.1, 32.0)	27.3 (25.3, 29.4)	32.2 (30.5, 33.9)
RMST in month (SD)				
1‐year	9.60 (0.04)	9.76 (0.04)	11.48 (0.03)	10.90 (0.05)
	−0.35 (−0.46, −0.23)**	Referent	0.49 (0.40, 0.57)**	Referent
2‐year	15.24 (0.09)	16.52 (0.10)	19.94 (0.13)	18.85 (0.14)
	−1.75 (−2.02, −1.48)**	Referent	0.82 (0.53, 1.12)**	Referent
5‐year	24.19 (0.23)	29.76 (0.27)	35.00 (0.48)	34.68 (0.44)
	−6.60 (−7.31, −5.89)**	Referent	−0.22 (−1.17, 0.73)	Referent

*Note:* Adjusted long‐term survival rates were derived from the propensity score inverse probability of treatment‐weighted cohort. RMST analyses were conducted in the unweighted original cohort, and differences between treatment arms were evaluated using multivariable RMST regression adjusting for baseline factors including age at diagnosis, race/ethnicity, health insurance, neighborhood median household income, urban/rural location, year of cancer diagnosis, comorbidity score, facility location, facility type, cancer stage, histologic type, and tumor grade.

Abbreviations: CI, confidence interval; RMST, restricted mean survival time; SD, standard deviation.

** *p* < 0.0001.

In a series of stratified analyses these findings were largely similar. Among patients < 70 years of age with no comorbidities, the survival was similar from diagnosis until 5 months after which time mortality was higher among patients treated with NACT (aHR = 1.67; 95% CI, 1.58–1.77) (Figure S2; Tables S2–S4). In the PP analysis in this cohort, 5‐year survival was 30.8% (95% CI, 28.2%–33.6%) for NACT compared to 36.9% (95% CI, 34.8%–39.2%) for PDS (Table S3). Similar findings were noted for both stage IVA and IVB tumors when stratified by stage (Tables S2–S4; Figure S3) and for endometrioid cancers, serous cancers and carcinosarcomas when stratified by histology (Tables S2–S4; Figure S4).

## 4 Discussion

These data suggest that use of NACT for advanced stage endometrial cancer has increased substantially in recent years. Patients treated with NACT have lower rates of short‐term mortality. For patients who survive the initial period after diagnosis, primary cytoreductive surgery is associated with better long‐term survival.

The utilization of NACT among patients with stage IV endometrial cancer has increased significantly from 30.3% in 2010 to 73.8% in 2021. In our cohort, nearly three quarters of patients were treated with NACT in 2021. The increase in use of NACT is likely driven by multiple factors [6]. First, although prospective data are lacking, studies have begun to suggest that NACT may be an appropriate treatment option for metastatic endometrial cancer [5, 6, 7, 8, 14, 15, 16, 17, 18, 19]. Second, there is now an abundance of evidence that ovarian cancer survival is similar for NACT and PDS. The increased use of NACT for ovarian cancer may have fueled similar interest for endometrial cancer [20, 21, 22, 23, 24, 25]. Finally, given that carcinomatosis is frequent among patients with stage IV endometrial cancer, undertaking primary surgery is technically demanding and associated with substantial morbidity. Meanwhile, use of NACT is associated with decreased need for radical surgery and lower morbidity [6, 26].

The relationship between primary therapy and survival for stage IV endometrial cancer is complex. We consistently noted that survival associated with NACT was similar to or better than for PDS in the first few months following diagnosis, but for those who survive initial therapy, survival for PDS is superior. In our PP analysis of patients who undertook both surgery and chemotherapy, the crossing of survival curves occurred two years after diagnosis. These findings suggest that immediate morbidity and mortality are high for patients treated with a strategy of primary cytoreduction. This is consistent with prior studies that have shown a decrease in operative time and transfusion rates for NACT patients compared to PDS patients without affecting optimal cytoreduction rates [15]. However, for those patients who survive initial therapy, there may be long‐term benefits to primary cytoreduction.

These findings are consistent with our prior work examining survival outcomes of utilization of NACT among patients with stage IV endometrial cancer [6, 26]. A recent systematic review that included nine studies examining NACT for metastatic endometrial cancer included more than 5000 patients of whom 23% received NACT as initial treatment. Pooled data demonstrated an 82% optimal cytoreduction rate for NACT followed by surgery compared to 52% for PDS. Median overall survival varied from 12 to 27 months among patients receiving NACT and from 18 to 25 months for among those undergoing PDS; however, these differences did not reach statistical significance. The perioperative morbidity for NACT was also lower compared to PDS [26]. These findings suggest that the survival rates remain low despite various treatment modalities, indicating the aggressive nature of stage IV endometrial cancer [26]. Notably, the largest study in the meta‐analysis also was based on patients derived from the NCDB [6].

An important concern for the utilization of NACT for patients diagnosed with metastatic endometrial cancer is the possibility that patients will progress during initial chemotherapy and not have the opportunity to undergo cytoreductive surgery. In our cohort, 59% of patients in the NACT group began the treatments with chemotherapy but without undergoing surgery. A similar concern for patients who undergo PDS is that extensive surgery and the accompanying morbidity may preclude chemotherapy. In our cohort, 30% of patients who underwent PDS did not initiate chemotherapy. An important limitation of observational data is that we are unable to distinguish patients who initiate NACT with the intent of ultimately undergoing surgery versus those who are receiving palliative intent chemotherapy. In the systematic review, interval debulking surgery was an inclusion criterion in three out of the nine studies analyzed [7, 15, 19]. Excluding patients from those three studies, 41% of NACT‐treated patients did not undergo subsequent surgery [26]. In our stratified analysis in which we included patients < 70 years of age without comorbidity, a cohort more likely to undergo more aggressive therapy, our overall findings were similar to the primary cohort.

For patients who undergo surgery, whether performed as primary or interval surgery, the goal is to optimize cytoreduction to no gross residual disease (NGRD). A recent meta‐analysis reported that stage IV endometrial cancer patients who achieved NGRD or optimal status (< 1 cm) through maximal cytoreduction experienced better progression‐free and overall survival [3]. In this report, NGRD was achieved in 50% of the cases. Patients who underwent suboptimal cytoreduction (> 1 cm) had an elevated risk of recurrence and mortality. Any gross residual disease was linked to poorer progression‐free and overall survival [3]. When exploring ways to reduce surgical morbidity for stage IV endometrial cancer, recent studies suggest that minimally invasive surgery may be feasible for patients treated with NACT [27].

We acknowledge several important limitations. First, selection bias in treatment allocation is likely. Although PS methods were used to account for measured confounders, the possibility of unmeasured confounding remains. Second, we cannot distinguish NACT with the intent to undergo surgery from palliative chemotherapy. A series of stratified analyses were conducted to attempt to address this limitation. Third, we lack data on a few important oncologic variables including molecular tumor characteristics including the status of the mismatch repair proteins, response to treatment, cytoreductive status, recurrence rates, and cause of death, which would have provided additional insights. Lastly, although the NCDB captures a substantial proportion of patients with endometrial cancer, our findings may not be fully generalizable to all individuals or populations with advanced‐stage endometrial cancer.

In summary, the adoption of NACT for stage IV endometrial has grown in recent years, making primary chemotherapy the most frequent initial approach for newly diagnosed metastatic disease. A time varying association between the utilization of NACT and survival suggests short‐term, primary cytoreduction yields superior survival among patients who live longer. These findings highlight the importance of tailoring treatment decisions to individual patients with newly diagnosed stage IV endometrial cancer.

## Author Contributions

**Dib Sassine:** investigation (supporting), writing – review and editing (supporting). **Yongmei Huang:** formal analysis (lead), investigation (supporting), methodology (supporting), writing – original draft (supporting), writing – review and editing (supporting). **Chin Hur:** investigation (supporting), writing – review and editing (supporting). **Elena B. Elkin:** investigation (supporting), writing – review and editing (supporting). **Jennifer S. Ferris:** investigation (supporting), writing – review and editing (supporting). **Alex Melamed:** investigation (supporting), writing – review and editing (supporting). **Chung Yin Kong:** investigation (supporting), writing – review and editing (supporting). **Evan R. Myers:** investigation (supporting), writing – review and editing (supporting). **Nina A. Bickell:** investigation (supporting), writing – review and editing (supporting). **William D. Hazelton:** investigation (supporting), writing – review and editing (supporting). **Tracy M. Layne:** investigation (supporting), writing – review and editing (supporting). **Brandy Heckman‐Stoddard:** investigation (supporting), writing – review and editing (supporting). **Goli Samimi:** investigation (supporting), writing – review and editing (supporting). **Laura J. Havrilesky:** investigation (supporting), writing – review and editing (supporting). **Stephanie V. Blank:** investigation (supporting), writing – review and editing (supporting). **Xiao Xu:** investigation (supporting), writing – review and editing (supporting). **Jason D. Wright:** formal analysis (supporting), funding acquisition (lead), investigation (lead), methodology (supporting), writing – original draft (supporting).

## Funding

This work was supported by the National Cancer Institute 1U01 CA265739 to Jason D. Wright.

## Conflicts of Interest

Dr. Wright has received royalties from UpToDate, honoraria from the American College of Obstetricians and Gynecologists, and research support from Merck. Dr. Xu has received honoraria from the American Association of Gynecologic Laparoscopists. Dr. Elkin has received research support from Pfizer. The other authors have no conflicts of interest.

## Supporting information


**Data S1:** cam471539‐sup‐0001‐Figures.pptx.

**Data S2:** cam471539‐sup‐0002‐Tables.docx.

## Data Availability

Data is publicly available.
