# Supplementary material for: Neoadjuvant Chemotherapy Versus Primary Cytoreductive Surgery for Metastatic Endometrial Cancer
Source: Cancer Med. 2026 Jan 20;15(1):e71539. doi: 10.1002/cam4.71539 (PMC12819165; doi:10.1002/cam4.71539)
Supplement: Supplementary file 1 — Data S1: cam471539‐sup‐0001‐Figures.pptx. [file CAM4-15-e71539-s002.pptx]

## Slide 1
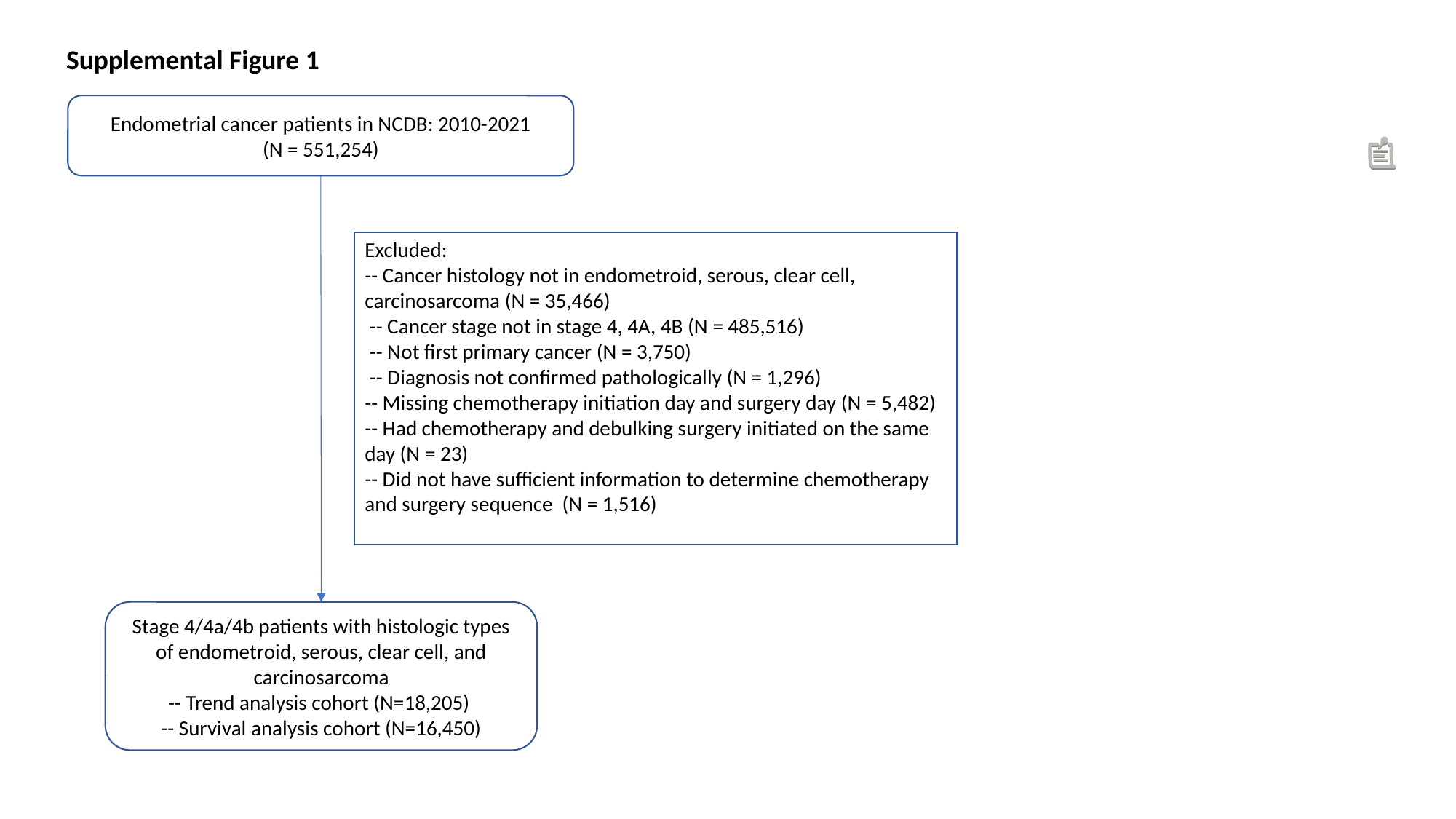

Supplemental Figure 1
Endometrial cancer patients in NCDB: 2010-2021
(N = 551,254)
Stage 4/4a/4b patients with histologic types of endometroid, serous, clear cell, and carcinosarcoma
-- Trend analysis cohort (N=18,205)
-- Survival analysis cohort (N=16,450)
Excluded:
-- Cancer histology not in endometroid, serous, clear cell, carcinosarcoma (N = 35,466)
 -- Cancer stage not in stage 4, 4A, 4B (N = 485,516)
 -- Not first primary cancer (N = 3,750)
 -- Diagnosis not confirmed pathologically (N = 1,296)
-- Missing chemotherapy initiation day and surgery day (N = 5,482)
-- Had chemotherapy and debulking surgery initiated on the same day (N = 23)
-- Did not have sufficient information to determine chemotherapy and surgery sequence (N = 1,516)

## Slide 2
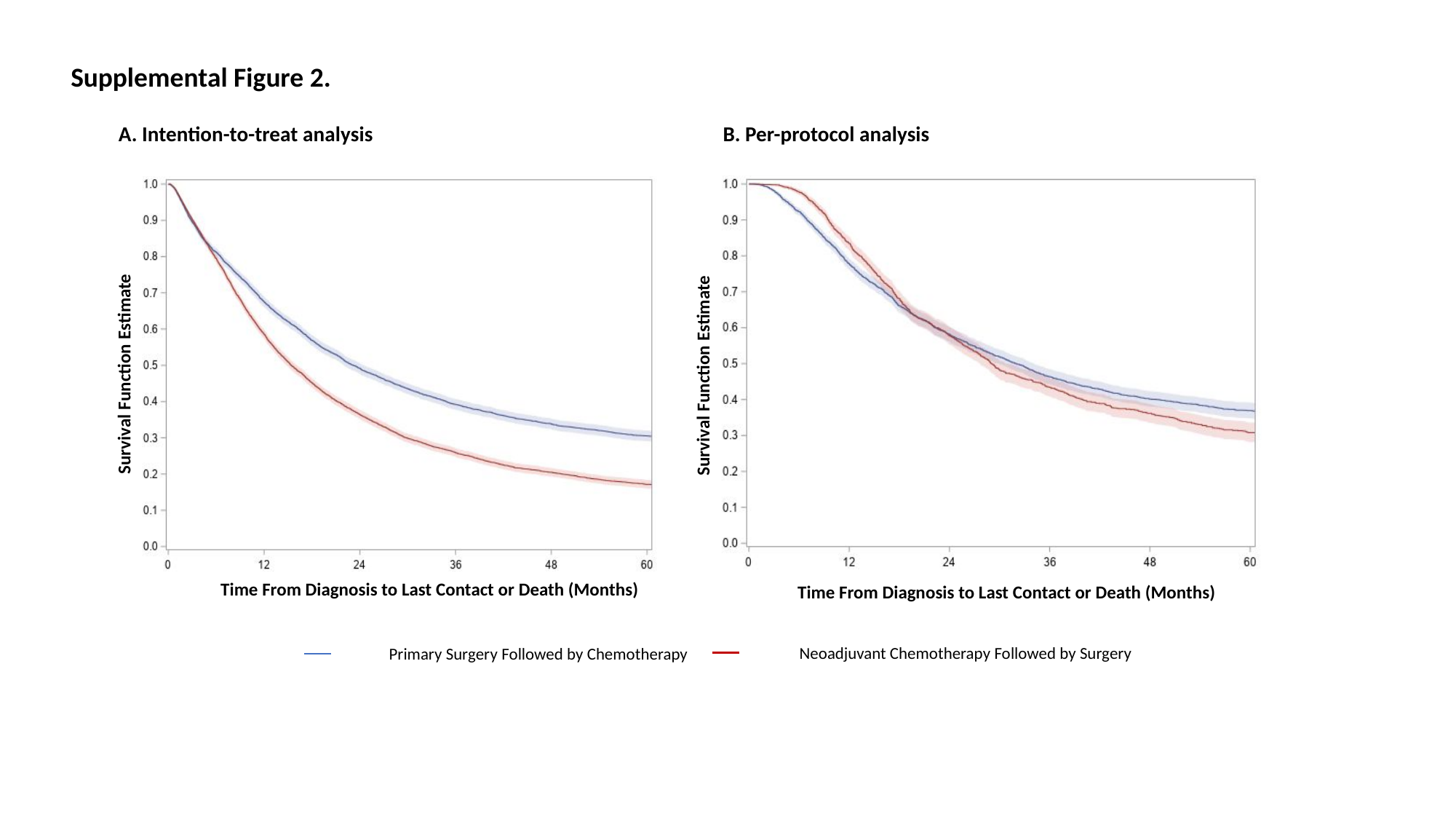

Supplemental Figure 2.
A. Intention-to-treat analysis
B. Per-protocol analysis
Survival Function Estimate
Time From Diagnosis to Last Contact or Death (Months)
Survival Function Estimate
Time From Diagnosis to Last Contact or Death (Months)
Neoadjuvant Chemotherapy Followed by Surgery
Primary Surgery Followed by Chemotherapy

## Slide 3
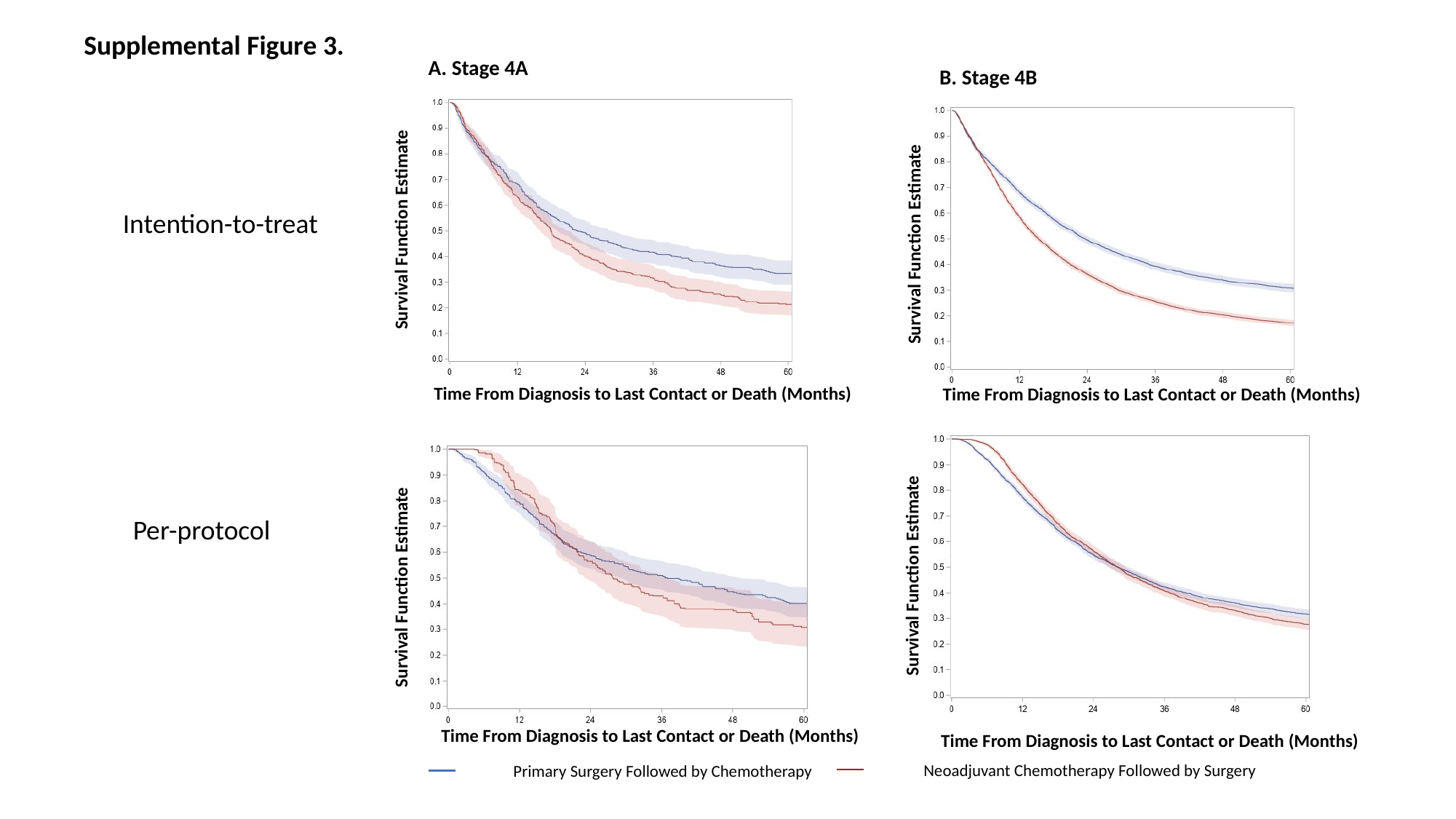

Supplemental Figure 3.
A. Stage 4A
B. Stage 4B
Intention-to-treat
Survival Function Estimate
Survival Function Estimate
Time From Diagnosis to Last Contact or Death (Months)
Time From Diagnosis to Last Contact or Death (Months)
Per-protocol
Survival Function Estimate
Survival Function Estimate
Time From Diagnosis to Last Contact or Death (Months)
Time From Diagnosis to Last Contact or Death (Months)
Neoadjuvant Chemotherapy Followed by Surgery
Primary Surgery Followed by Chemotherapy

## Slide 4
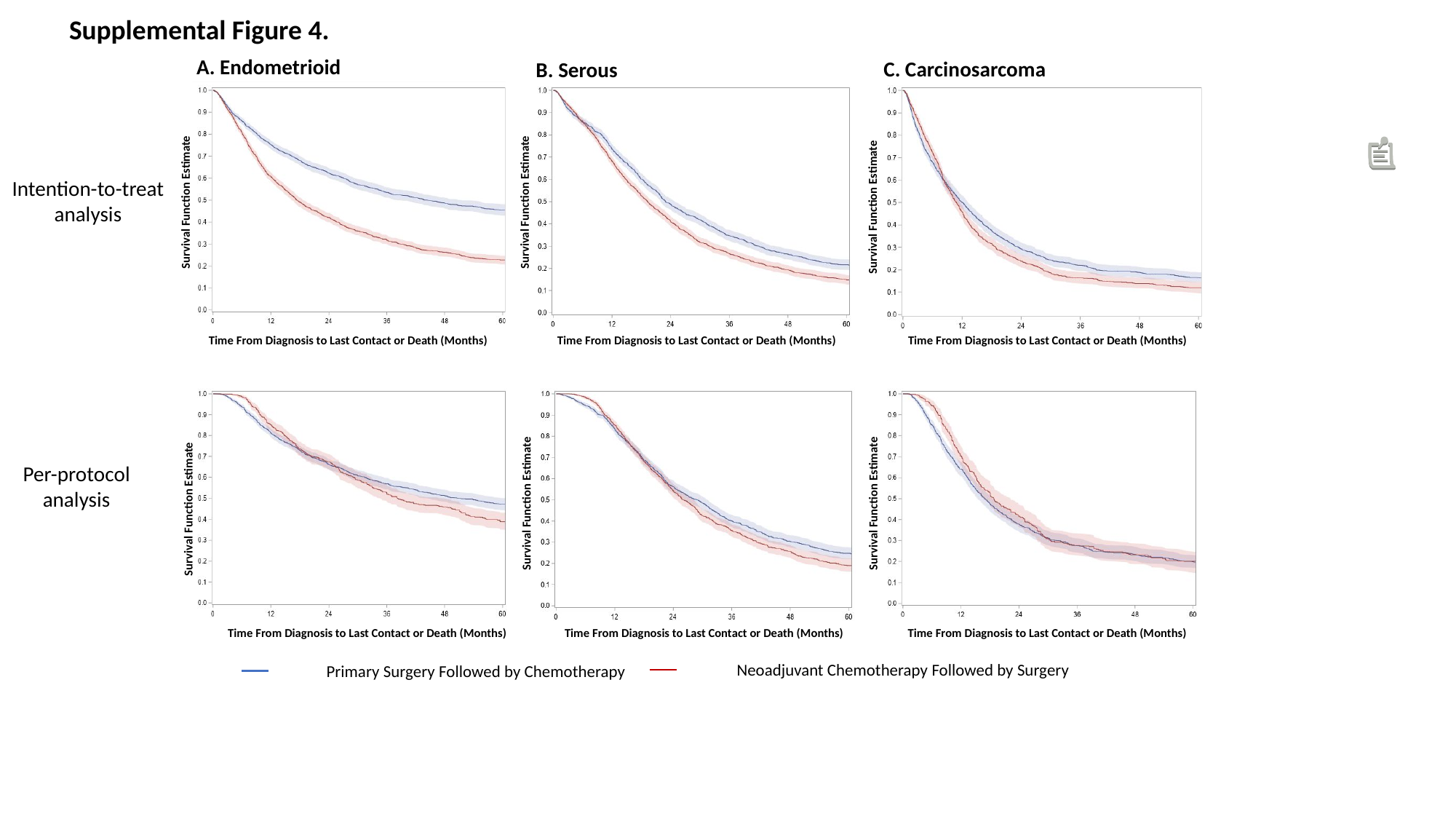

Supplemental Figure 4.
A. Endometrioid
C. Carcinosarcoma
B. Serous
Intention-to-treat
analysis
Survival Function Estimate
Survival Function Estimate
Survival Function Estimate
Time From Diagnosis to Last Contact or Death (Months)
Time From Diagnosis to Last Contact or Death (Months)
Time From Diagnosis to Last Contact or Death (Months)
Per-protocol
analysis
Survival Function Estimate
Survival Function Estimate
Survival Function Estimate
Time From Diagnosis to Last Contact or Death (Months)
Time From Diagnosis to Last Contact or Death (Months)
Time From Diagnosis to Last Contact or Death (Months)
Neoadjuvant Chemotherapy Followed by Surgery
Primary Surgery Followed by Chemotherapy
