# Supplementary material for: Neoadjuvant Chemotherapy Versus Primary Cytoreductive Surgery for Metastatic Endometrial Cancer
Source: Cancer Med. 2026 Jan 20;15(1):e71539. doi: 10.1002/cam4.71539 (PMC12819165; doi:10.1002/cam4.71539)
Supplement: Supplementary file 2 — Data S2: cam471539‐sup‐0002‐Tables.docx. [file CAM4-15-e71539-s001.docx]

Supplemental Table 1. Balance diagnostics for demographics and clinical factors in original unadjusted intention-to-treat cohort and per-protocol cohort.

|  | **Intention-to-treat analysis** | | |  | **Per-protocol analysis** | | |
| --- | --- | --- | --- | --- | --- | --- | --- |
|  | **Primary surgery** | **Neoadjuvant chemotherapy** | **SMD** |  | **Primary surgery followed by chemotherapy** | **Neoadjuvant chemotherapy followed by surgery** | **SMD** |
|  | **N (%)** | **N (%)** |  |  | **N (%)** | **N (%)** |  |
| **Total** | 7,603 (100%) | 8,847 (100%) |  |  | 4,772 (100%) | 3,541 (100%) |  |
| **Age of Diagnosis** |  |  | 0.10 |  |  |  | 0.17 |
| <40 | 165 (2.2) | 223 (2.5) |  |  | 109 (2.3) | 91 (2.6) |  |
| 40-49 | 463 (6.1) | 504 (5.7) |  |  | 305 (6.4) | 211 (6.0) |  |
| 50-59 | 1,687 (22.2) | 1,887 (21.3) |  |  | 1,106 (23.2) | 748 (21.1) |  |
| 60-69 | 3,010 (39.6) | 3,634 (41.1) |  |  | 2,015 (42.2) | 1,549 (43.7) |  |
| 70-79 | 1,685 (22.2) | 2,050 (23.2) |  |  | 989 (20.7) | 802 (22.6) |  |
| >/= 80 | 593 (7.8) | 549 (6.2) |  |  | 248 (5.2) | 140 (4.0) |  |
| **Race/Ethnicity** |  |  | 0.16 |  |  |  | 0.11 |
| Non-Hispanic: White | 5,041 (66.3) | 5,570 (63.0) |  |  | 3,149 (66.0) | 2,301 (65.0) |  |
| Non-Hispanic: Black | 1,446 (19.0) | 1,979 (22.4) |  |  | 902 (18.9) | 715 (20.2) |  |
| Non-Hispanic: Other | 403 (5.3) | 405 (4.6) |  |  | 274 (5.7) | 187 (5.3) |  |
| Hispanic | 477 (6.3) | 691 (7.8) |  |  | 310 (6.5) | 278 (7.9) |  |
| Unknown | 236 (3.1) | 202 (2.3) |  |  | 137 (2.9) | 60 (1.7) |  |
| **Year of Diagnosis** |  |  | 0.48 |  |  |  | 0.58 |
| 2010 | 938 (12.3) | 408 (4.6) |  |  | 488 (10.2) | 142 (4) |  |
| 2011 | 796 (10.5) | 410 (4.6) |  |  | 445 (9.3) | 145 (4.1) |  |
| 2012 | 793 (10.4) | 501 (5.7) |  |  | 490 (10.3) | 171 (4.8) |  |
| 2013 | 776 (10.2) | 600 (6.8) |  |  | 492 (10.3) | 188 (5.3) |  |
| 2014 | 707 (9.3) | 688 (7.8) |  |  | 481 (10.1) | 247 (7) |  |
| 2015 | 659 (8.7) | 750 (8.5) |  |  | 443 (9.3) | 299 (8.4) |  |
| 2016 | 643 (8.5) | 918 (10.4) |  |  | 433 (9.1) | 341 (9.6) |  |
| 2017 | 605 (8) | 1,007 (11.4) |  |  | 395 (8.3) | 401 (11.3) |  |
| 2018 | 566 (7.4) | 1,098 (12.4) |  |  | 370 (7.8) | 477 (13.5) |  |
| 2019 | 622 (8.2) | 1,214 (13.7) |  |  | 412 (8.6) | 548 (15.5) |  |
| 2020 | 498 (6.6) | 1,253 (14.2) |  |  | 323 (6.8) | 582 (16.4) |  |
| **Insurance status** |  |  | 0.14 |  |  |  | 0.21 |
| Uninsured | 333 (4.4) | 432 (4.9) |  |  | 206 (4.3) | 135 (3.8) |  |
| Private | 3,048 (40.1) | 3,240 (36.6) |  |  | 2,022 (42.4) | 1436 (40.6) |  |
| Medicaid | 601 (7.9) | 976 (11.0) |  |  | 386 (8.1) | 339 (9.6) |  |
| Medicare | 3,415 (44.9) | 3,953 (44.7) |  |  | 2,030 (42.5) | 1549 (43.7) |  |
| Other Government | 67 (0.9) | 84 (0.9) |  |  | 46 (1.0) | 41 (1.2) |  |
| Unknown | 139 (1.8) | 162 (1.8) |  |  | 82 (1.7) | 41 (1.2) |  |
| **Neighborhood median household income** |  |  | 0.06 |  |  |  | 0.08 |
| < $46,277 | 1,178 (15.5) | 1,527 (17.3) |  |  | 707 (14.8) | 564 (15.9) |  |
| $46,277 - $57,856 | 1,384 (18.2) | 1,570 (17.7) |  |  | 878 (18.4) | 619 (17.5) |  |
| $57,857 - $74,062 | 1,702 (22.4) | 1,816 (20.5) |  |  | 1,057 (22.2) | 706 (19.9) |  |
| $74,063 + | 2,359 (31.0) | 2,662 (30.1) |  |  | 1,504 (31.5) | 1,119 (31.6) |  |
| Unknown | 980 (12.9) | 1,272 (14.4) |  |  | 626 (13.1) | 533 (15.1) |  |
| **Urban/Rural** |  |  | 0.00 |  |  |  | 0.00 |
| Metropolitan | 6,296 (82.8) | 7,372 (83.3) |  |  | 3,959 (83) | 2,937 (82.9) |  |
| Urban | 924 (12.2) | 1,054 (11.9) |  |  | 573 (12) | 417 (11.8) |  |
| Rural | 130 (1.7) | 136 (1.5) |  |  | 77 (1.6) | 61 (1.7) |  |
| Unknown | 253 (3.3) | 285 (3.2) |  |  | 163 (3.4) | 126 (3.6) |  |
| **Facility location** |  |  | 0.14 |  |  |  | 0.12 |
| Northeast | 1,618 (21.3) | 2,100 (23.7) |  |  | 1,047 (21.9) | 800 (22.6) |  |
| South | 2,179 (28.7) | 2,131 (24.1) |  |  | 1,318 (27.6) | 874 (24.7) |  |
| Midwest | 2,423 (31.9) | 3,052 (34.5) |  |  | 1,603 (33.6) | 1,171 (33.1) |  |
| West | 1,218 (16) | 1,341 (15.2) |  |  | 695 (14.6) | 605 (17.1) |  |
| Unknown | 165 (2.2) | 223 (2.5) |  |  | 109 (2.3) | 91 (2.6) |  |
| **Facility type** |  |  | 0.15 |  |  |  | 0.13 |
| Community Cancer Program | 261 (3.4) | 350 (4.0) |  |  | 125 (2.6) | 85 (2.4) |  |
| Comprehensive Community Cancer Program | 2,611 (34.3) | 2,645 (29.9) |  |  | 1,520 (31.9) | 991 (28) |  |
| Academic/Research Program | 3,006 (39.5) | 4,014 (45.4) |  |  | 2,019 (42.3) | 1,622 (45.8) |  |
| Integrated Network Cancer Program | 1,560 (20.5) | 1,615 (18.3) |  |  | 999 (20.9) | 752 (21.2) |  |
| Other or unknown | 165 (2.2) | 223 (2.5) |  |  | 109 (2.3) | 91 (2.6) |  |
| **Cancer stage** |  |  | 0.16 |  |  |  | 0.16 |
| IV NOS | 507 (6.7) | 385 (4.4) |  |  | 323 (6.8) | 141 (4) |  |
| IVA | 665 (8.7) | 615 (7.0) |  |  | 416 (8.7) | 246 (6.9) |  |
| IVB | 6,431 (84.6) | 7,847 (88.7) |  |  | 4,033 (84.5) | 3,154 (89.1) |  |
| **Histology type** |  |  | 0.26 |  |  |  | 0.28 |
| Endometrioid | 2,711 (35.7) | 2,698 (30.5) |  |  | 1,583 (33.2) | 956 (27) |  |
| Serous | 1,903 (25.0) | 2,660 (30.1) |  |  | 1,342 (28.1) | 1,450 (40.9) |  |
| Clear cell | 297 (3.9) | 314 (3.5) |  |  | 205 (4.3) | 127 (3.6) |  |
| Carcinosarcoma | 1,500 (19.7) | 1,204 (13.6) |  |  | 894 (18.7) | 481 (13.6) |  |
| EM NOS | 1,192 (15.7) | 1,971 (22.3) |  |  | 748 (15.7) | 527 (14.9) |  |
| **Tumor grade** |  |  | 0.46 |  |  |  | 0.33 |
| Well | 460 (6.1) | 399 (4.5) |  |  | 199 (4.2) | 143 (4) |  |
| Moderate | 869 (11.4) | 809 (9.1) |  |  | 538 (11.3) | 302 (8.5) |  |
| Poorly | 3,803 (50.0) | 3,138 (35.5) |  |  | 2,447 (51.3) | 1,405 (39.7) |  |
| Undifferentiated | 750 (9.9) | 657 (7.4) |  |  | 503 (10.5) | 321 (9.1) |  |
| Unknown | 1,721 (22.6) | 3,844 (43.4) |  |  | 1,085 (22.7) | 1,370 (38.7) |  |
| **Charlson comorbidity score** |  |  | 0.08 |  |  |  | 0.09 |
| 0 | 5,615 (73.9) | 6,483 (73.3) |  |  | 3,497 (73.3) | 2,671 (75.4) |  |
| 1 | 1,526 (20.1) | 1,647 (18.6) |  |  | 977 (20.5) | 614 (17.3) |  |
| 2 | 297 (3.9) | 464 (5.2) |  |  | 192 (4.0) | 173 (4.9) |  |
| >=3 | 165 (2.2) | 253 (2.9) |  |  | 106 (2.2) | 83 (2.3) |  |

SMD: Standardized mean difference

Supplemental Table 2. Adjusted short-term mortality in sub-group analyses.

|  | **IPTW Intend-to-treat Cohort** | |  | **IPTW Per-protocol Cohort** | |
| --- | --- | --- | --- | --- | --- |
|  | **Neoadjuvant chemotherapy**  **% (95%CI)** | **Primary surgery**  **% (95%CI)** |  | **Neoadjuvant chemotherapy followed by surgery**  **% (95%CI)** | **Primary surgery followed by chemotherapy**  **% (95%CI)** |
| **<70 yrs, no comorbidity** |  |  |  |  |  |
| 30-day | 1.5 (1.1, 1.9) | 1.9 (1.4, 2.4) |  | 0.1 (0.0, 0.2) | 0.1 (0.0, 0.2) |
| 60-day | 5.4 (4.7, 6.1) | 6.0 (5.2, 6.9) |  | 0.1 (0.0, 0.3) | 0.7 (0.4, 1.0) |
| 90-day | 9.3 (8.3, 10.3) | 9.3 (8.3, 10.4) |  | 0.2 (0.0, 0.4) | 1.9 (1.3, 2.4) |
| 120-day | 12.6 (11.4, 13.7) | 12.4 (11.1, 13.6) |  | 0.6 (0.2, 1.0) | 3.9 (3.1, 4.7) |
| 180-day | 19.7 (18.3, 21.2) | 16.6 (15.2, 18.0) |  | 2.2 (1.4, 3.0) | 7.7 (6.6, 8.8) |
| **Stage 4A** |  |  |  |  |  |
| 30-day | 1.4 (0.4, 2.3) | 1.6 (0.5, 2.7) |  | 0.0 (0.0, 0.0) | 0.2 (0.0, 0.7) |
| 60-day | 5.4 (3.4, 7.4) | 7.5 (4.9, 10.1) |  | 0.0 (0.0, 0.0) | 1.8 (0.6, 2.9) |
| 90-day | 10.4 (7.7, 13.0) | 11.5 (8.5, 14.4) |  | 0.0 (0.0, 0.0) | 3.4 (1.7, 5.1) |
| 120-day | 13.0 (9.9, 15.9) | 13.9 (10.7, 17.1) |  | 0.0 (0.0, 0.0) | 4.0 (2.2, 5.9) |
| 180-day | 18.7 (15.1, 22.2) | 19.8 (16.1, 23.4) |  | 1.4 (0.1, 2.7) | 8.6 (5.9, 11.3) |
| **Stage 4B** |  |  |  |  |  |
| 30-day | 1.8 (1.4, 2.1) | 2.1 (1.7, 2.5) |  | 0.1 (0.0, 0.2) | 0.1 (0.0, 0.2) |
| 60-day | 5.9 (5.3, 6.6) | 6.3 (5.6, 7.1) |  | 0.2 (0.0, 0.3) | 0.8 (0.5, 1.1) |
| 90-day | 9.7 (8.8, 10.5) | 10.4 (9.4, 11.3) |  | 0.2 (0.0, 0.4) | 2.0 (1.5, 2.4) |
| 120-day | 13.3 (12.3, 14.2) | 14.0 (12.9, 15.1) |  | 0.6 (0.2, 1.0) | 4.2 (3.5, 4.8) |
| 180-day | 20.6 (19.4, 21.7) | 18.7 (17.4, 19.9) |  | 2.2 (1.5, 2.9) | 8.0 (7.2, 8.8) |
| **Endometroid** |  |  |  |  |  |
| 30-day | 1.3 (0.9, 1.8) | 1.5 (1.0, 1.9) |  | 0.0 (0.0, 0.0) | 0.1 (0.0, 0.1) |
| 60-day | 5.0 (4.0, 6.0) | 4.3 (3.4, 5.2) |  | 0.1 (0.0, 0.4) | 0.3 (0.1, 0.6) |
| 90-day | 8.7 (7.4, 10.0) | 7.4 (6.3, 8.5) |  | 0.1 (0.0, 0.4) | 1.4 (0.7, 2.0) |
| 120-day | 11.7 (10.2, 13.1) | 10.3 (9.0, 11.6) |  | 0.3 (0.0, 0.6) | 3.1 (2.2, 4.0) |
| 180-day | 19.1 (17.3, 20.8) | 14.1 (12.5, 15.6) |  | 1.4 (0.5, 2.2) | 6.2 (5.0, 7.5) |
| **Serous** |  |  |  |  |  |
| 30-day | 1.2 (0.8, 1.7) | 1.2 (0.7, 1.7) |  | 0.0 (0.0, 0.0) | 0.2 (0.0, 0.5) |
| 60-day | 4.2 (3.4, 5.0) | 5.0 (3.9, 6.0) |  | 0.0 (0.0, 0.0) | 1.0 (0.4, 1.5) |
| 90-day | 6.5 (5.5, 7.5) | 8.3 (6.8, 9.7) |  | 0.0 (0.0, 0.1) | 1.8 (1.2, 2.5) |
| 120-day | 8.8 (7.6, 10.0) | 10.7 (9.1, 12.2) |  | 0.3 (0.0, 0.6) | 3.2 (2.3, 4.1) |
| 180-day | 14.3 (12.7, 15.8) | 13.9 (12.2, 15.7) |  | 1.6 (0.9, 2.3) | 5.3 (4.1, 6.5) |
| **Clear cell** |  |  |  |  |  |
| 30-day | 2.9 (0.9, 4.9) | 2.6 (0.8, 4.3) |  | 0.0 (0.0, 0.0)* | 0.0 (0.0, 0.0)* |
| 60-day | 8.1 (4.4, 11.6) | 5.2 (2.6, 7.7) |  | 0.0 (0.0, 0.0)* | 0.5 (0.1,3.4)* |
| 90-day | 11.4 (7.3, 15.4) | 8.1 (4.7, 11.4) |  | 0.0 (0.0, 0.0)* | 1.5 (0.5,4.5)* |
| 120-day | 13.8 (9.4, 18.0) | 14.7 (9.6, 19.6) |  | 0.0 (0.0, 0.0)* | 5.9 (3.4,10.1)* |
| 180-day | 22.7 (16.9, 28.1) | 22.9 (16.9, 28.6) |  | 1.6 (0.4,6.1)* | 13.7 (9.6,19.2)* |
| **Carcinosarcoma** |  |  |  |  |  |
| 30-day | 2.3 (1.3, 3.2) | 3.6 (2.6, 4.5) |  | 0.0 (0.0, 0.0) | 0.0 (0.0, 0.0) |
| 60-day | 7.9 (6.1, 9.7) | 11.8 (9.9, 13.6) |  | 0.2 (0.0, 0.6) | 1.6 (0.8, 2.4) |
| 90-day | 13.3 (11.1, 15.5) | 18.0 (15.8, 20.2) |  | 0.7 (0.0, 1.5) | 3.7 (2.4, 5.0) |
| 120-day | 18.2 (15.6, 20.6) | 23.0 (20.4, 25.4) |  | 1.8 (0.4, 3.2) | 6.8 (4.9, 8.6) |
| 180-day | 27.4 (24.5, 30.1) | 31.9 (29.2, 34.6) |  | 5.6 (3.0, 8.2) | 14.8 (12.4, 17.2) |

*Propensity score model for clear cell cohort did not converge because of small sample size. We reported observed survival. CI: Confidence Interval

Supplemental Table 3. Adjusted long-term survival in sub-group analyses.

|  | **Intention-to-treat analysis** | |  | **Per-protocol analysis** | |
| --- | --- | --- | --- | --- | --- |
|  | **Neoadjuvant chemotherapy**  **% (95%CI)** | **Primary**  **surgery**  **% (95%CI)** |  | **Neoadjuvant chemotherapy followed by surgery**  **% (95%CI)** | **Primary surgery followed by chemotherapy**  **% (95%CI)** |
| **<70 yrs, no comorbidity** |  |  |  |  |  |
| 1-year | 60.2 (58.6, 61.9) | 70.2 (68.6, 72.0) |  | 83.4 (81.6, 85.4) | 77.8 (76.2, 79.4) |
| 2-year | 38.5 (36.9, 40.2) | 53.3 (51.5, 55.2) |  | 57.8 (55.4, 60.4) | 58.2 (56.2, 60.3) |
| 5-year | 19.8 (18.3, 21.4) | 35.6 (33.7, 37.6) |  | 30.8 (28.2, 33.6) | 36.9 (34.8, 39.2) |
| **Stage 4A** |  |  |  |  |  |
| 1-year | 62.9 (58.4, 67.7) | 68.1 (63.7, 72.7) |  | 84.0 (77.9, 90.6) | 79.2 (75.3, 83.2) |
| 2-year | 40.1 (35.6, 45.2) | 49.3 (44.8, 54.3) |  | 56.5 (49.5, 64.5) | 58.8 (53.7, 64.4) |
| 5-year | 21.2 (17.1, 26.3) | 33.5 (29.0, 38.6) |  | 30.7 (23.4, 40.3) | 40.2 (34.7, 46.6) |
| **Stage 4B** |  |  |  |  |  |
| 1-year | 58.4 (57.1, 59.7) | 67.9 (66.4, 69.5) |  | 82.2 (80.5, 83.8) | 77.2 (75.9, 78.6) |
| 2-year | 36.2 (34.9, 37.5) | 49.6 (48.0, 51.2) |  | 56.2 (54.1, 58.4) | 54.9 (53.2, 56.6) |
| 5-year | 17.2 (16.0, 18.5) | 30.8 (29.2, 32.5) |  | 27.7 (25.6, 29.9) | 31.7 (29.9, 33.6) |
| **Endometroid** |  |  |  |  |  |
| 1-year | 60.5 (58.5, 62.6) | 75.2 (73.2, 77.1) |  | 85.2 (82.4, 88.0) | 81.2 (79.2, 83.3) |
| 2-year | 42.0 (39.9, 44.2) | 62.3 (60.1, 64.6) |  | 67.4 (63.9, 71.2) | 66.2 (63.8, 68.8) |
| 5-year | 22.6 (20.7, 24.7) | 45.5 (43.0, 48.0) |  | 38.9 (35.0, 43.2) | 47.2 (44.3, 50.2) |
| **Serous** |  |  |  |  |  |
| 1-year | 68.1 (66.1, 70.2) | 73.7 (71.5, 76.0) |  | 85.3 (83.2, 87.4) | 83.4 (81.3, 85.5) |
| 2-year | 41.1 (38.8, 43.6) | 48.8 (46.3, 51.4) |  | 54.2 (51.1, 57.6) | 56.2 (53.4, 59.2) |
| 5-year | 14.7 (12.7, 17.0) | 21.6 (19.4, 24.1) |  | 18.9 (16.0, 22.3) | 24.8 (22.1, 27.7) |
| **Clear Cell** |  |  |  |  |  |
| 1-year | 58.4 (51.9, 65.7) | 64.0 (58.1, 70.6) |  | 78.7 (70.5,84.8)* | 68.0 (61.1,74.0)* |
| 2-year | 32.4 (26.7, 39.4) | 43.1 (37.0, 50.1) |  | 49.4 (40.3,57.9)* | 43.0 (36.0,49.7)* |
| 5-year | 20.1 (14.9, 27.1) | 26.7 (21.3, 33.6) |  | 30.0 (21.5,39.0)* | 27.2 (20.9,33.8)* |
| **Carcinosarcoma** |  |  |  |  |  |
| 1-year | 45.8 (42.9, 48.9) | 50.0 (47.0, 53.2) |  | 70.4 (65.9, 75.1) | 64.2 (60.8, 67.7) |
| 2-year | 23.7 (21.1, 26.7) | 29.3 (26.7, 32.2) |  | 41.6 (36.5, 47.3) | 37.6 (34.3, 41.3) |
| 5-year | 12.0 (9.8, 14.7) | 16.5 (14.4, 18.9) |  | 20.1 (15.7, 25.9) | 19.8 (17.0, 23.1) |

*Propensity score model for clear cell cohort did not converge because of small sample size. We reported observed survival. CI: Confidence Interval

Supplemental Table 4. Pair-wise adjusted hazard ratio in overall cohort and sub-groups.

| **Sub-group** | **Survival curve crossing** | **aHR (95%CI) before crossing**  **aHR (95%CI)** | **aHR (95%CI) after crossing**  **aHR (95%CI)** |
| --- | --- | --- | --- |
| **Intention-to-treat analysis** |  |  |  |
| Overall cohort | 4 months | 1.03 (0.96-1.11) | 1.58 (1.51-1.64) |
| <70 yrs, no comorbidity | 5 months | 1.06 (0.998-1.226) | 1.67 (1.58-1.77) |
| Stage 4A | 16 months | 1.16 (0.98-1.36) | 1.88 (1.52-2.32) |
| Stage 4B | 4 months | 1.03 (0.96-1.12) | 1.59 (1.52-1.66) |
| Endometroid | 3 months | 1.15 (0.99-1.34) | 2.02 (1.88-2.18) |
| Serous | 6 months | 1.08 (0.94-1.24) | 1.34 (1.25-1.45) |
| Clear cell |  | 1.24 (0.99-1.55) | |
| Carcinosarcoma | 13 months | 1.10 (0.997-1.22) | 1.17 (1.01-1.36) |
| **Per-protocol analysis** |  |  |  |
| Overall cohort | 24 months | 0.93 (0.88-0.99) | 1.34 (1.23-1.47) |
| <70 yrs, no comorbidity | 17 months | 0.89 (0.80-0.98) | 1.33 (1.20-1.47) |
| Stage 4A | 16 months | 0.70 (0.53-0.94) | 1.89 (1.45-2.47) |
| Stage 4B | 24 months | 0.93 (0.79-0.97) | 1.27 (1.15-1.40) |
| Endometroid | 14 months | 0.81 (0.69-0.96) | 1.42(1.24-1.63) |
| Serous | 8 months | 0.65 (0.51-0.83) | 1.23(1.12-1.34) |
| Carcinosarcoma | 17 months | 0.82 (0.70-0.96) | 1.23(0.997-1.51) |

aHR: Adjusted Hazard Ratio; CI: Confidence Interval

**Supplemental Figures:**

Supplemental Figure 1 Cohort selection flow chart

Supplemental Figure 2. Inverse probability treatment weighting adjusted survival curves for patients younger than 70 years without comorbidities

A. Intention-to-treat analysis; B. Per-protocol analysis

Supplemental Figure 3. Inverse probability treatment weighting adjusted survival curves by stage

A. Stage 4A; B. Stage 4B

Supplemental Figure 4. Inverse probability treatment weighting adjusted survival curves by histology

1. Endometrial; B. Serous; C. Carcinoma
